# COVID-19 pandemic: Direct effects on the medical education in Pakistan

**DOI:** 10.1016/j.amsu.2022.104073

**Published:** 2022-06-24

**Authors:** Haziq Siddiqi, Muhammad Junaid Tahir, Irfan Ullah, Abubakar Nazir, Zain Douba, Muhammad Sohaib Asghar, Zohaib Yousaf

**Affiliations:** University of California, San Francisco, United States; Lahore General Hospital, Lahore, 54000, Pakistan; Kabir Medical College, Gandhara University, Peshawar, Pakistan; King Edward Medical University, Lahore, Pakistan; Faculty of Medicine, University of Aleppo, Aleppo, Syria; CME Office, Faculty of Medicine, University of Aleppo, Aleppo, Syria; Dow University of Health Sciences–Ojha Campus, Karachi, Pakistan; Hamad Medical Corporation, Doha, Qatar

**Keywords:** SARS-CoV-2, Outbreak, Medical education, Learning, Resources, Telemedicine

## Abstract

• To date, the medical education literature has focused on strategies for high-income countries to optimize online education.
• Low- and middle-income countries' students face additional challenges in providing virtual education.
• The main challenges LMIC students face are frequent internet and electricity outages and limited technology skills.
• The COVID-19 pandemic placed novel emotional and financial pressures on medical students.
• Here, we presented strategies for best practices in remote medical education, such as open-access medical education content.

The coronavirus disease 2019 (COVID-19) pandemic has impacted multiple facets of medical education, ranging from the admissions process to influencing students’ specialty selection. Initially intended as temporary solutions, many institutions introduced online learning platforms that have persisted due to the unpredictable and evolving course of the pandemic. At the time of writing, online medical education is becoming a new norm that is projected to continue for years to come [1]. To date, the medical education literature has primarily focused on strategies for high-income countries (HICs) to optimize online medical education. By contrast, low- and middle-income countries (LMICs) face additional challenges in providing virtual education. These challenges include resource limitations, such as frequent internet and electricity outages, limited technology skills, and institutional challenges. In this paper, we focus on lessons learned from medical education in Pakistan. We discuss specific challenges and their potential solutions, precisely resource, receiver, and institutional challenges. Also, we discuss additional strategies, including the role of global resources in medical education.

One of the primary concerns of students is inconsistent internet access. Even in developed countries, poor internet connection has been described as a barrier to remote medical education [2]. In Pakistan, limited internet access is a significant obstacle to the successful implementation of live education. In one survey of 532 medical students in Pakistan, 14% reported not having experience with internet use, and 15% reported only rarely using the internet [3]. Strategies to address this should include institutional venues with reliable internet access and governmental and private sector efforts to improve connectivity.

Multiple areas in Pakistan are affected by frequent electricity outages. While wealthier families have backup electricity sources, many families in Pakistan lack the facility of alternative source. Particularly for live educational sessions, electricity outages can result in interruption of educational content. For students without reliable electricity access, institutions should offer computer access within socially distanced computer labs to ensure all students access uninterrupted curricular content.

The rapid transition to a new educational format has also posed challenges for students with limited experience with technology. One survey from before the COVID-19 pandemic found that only 34% of students considered themselves capable of searching for clinical information on academic databases such as PubMed [3]. The same study also identified statistically significant differences concerning gender and ability to use online databases. Institutions must be cognizant of differences in students' technological skills. Specific solutions include introducing technology skills into the curriculum for both remote learnings and online clinical referencing.

By nature, online platforms make hands-on training challenging to replicate. The literature raises concerns that online learning may significantly affect students' preparation for their clinical years [1]. Training for specific skills, such as physical exam maneuvers and anatomic education, are particularly affected by virtual training. For these skills, virtual reality (VR)-based simulations can be a helpful replacement. Haroon et al. described the successful use of manikins and VR-based devices for skills training in dental education in Pakistan [4].

Similarly, VR-based anatomy education may be a promising alternative for anatomy content [5]. Institutions should also consider blended curricular arrangements, virtual education, and socially distanced, in-person training for hands-on skills. Going forward, institutions should consider educational strategies such as VR to ensure competency in hands-on training.

There have also been concerns regarding the integrity of the virtual assessment process [6]. These include reports of students using handheld electronic devices to share answers during the assessment [7]. Institutions must be cognizant of these additional challenges in remote contexts. Strategies to address this include arranging for critical assessments to be held in person with socially distanced settings.

The COVID-19 pandemic placed novel emotional and financial pressures on medical students, many of whom unfortunately do not typically have access to mental health resources [8]. Before COVID-19, mental illness has been prevalent among Pakistani medical students, with 35% reporting suicidal ideation over the past year [9]. Remote learning during COVID-19 is associated with increased feelings of isolation and anxiety, significantly when students cannot interact with other students [10,11]. Globally, work-related stress associated with COVID-19 has been linked to suicide by healthcare providers [12]. All authors, who are at various stages of medical education in their careers, strongly believe that institutions should make mental health resources and counselors available to students’ top priority.

Here, we discuss specific additional considerations for strategies to improve remote medical education.

In this section, we want to emphasize. Multiple institutions have developed and shared open-access medical education content, especially since the start of COVID-19. In response to students' concerns about outdated curriculum, incorporating such open-access content can be a valuable supplement to students' learning experience. For example, Rothberg et al. described an instance of faculty incorporating publicly available content from multiple sources into a curriculum for medical student education about otolaryngology [13]. While some resources are paid (UWorld, Amboss etc), many organizations offer free educational resources. Two leading organizations in this space include Clinical Problem Solvers (clinicalproblemsolving.com) and the Global Medical Education Collaborative (gmecollab.org). While the quality of these resources is excellent, many students are unaware of these initiatives. We encourage both students and educators to consider supplementing content with relevant resources from organizations such as these.

As a result of COVID-19, telemedicine has grown exponentially worldwide. This poses an opportunity for students to obtain clinical skills remotely. In the past, Pakistani medical educators have advocated for telemedicine as it can reduce disease exposure [14]. Tele-simulation, or faculty-supervised standardized patient telehealth encounters, have been proposed in the literature as one way to build skills in the preclinical years remotely [15,16]. Of note, in a survey of medical students at UC San Diego School of Medicine in the United States (US), telemedicine was one of the highest-rated educational resources [17]. In Pakistan, we believe that telemedicine can be a valuable educational resource for building clinical communication skills when possible.

In online learning settings, education is often provided in a lecture format, resulting in limited student engagement and many students being distracted by other content. One nationwide survey of students in the United Kingdom (UK) concluded that one strategy for effective online medical education is using team-based/problem-based learning, in which students discuss problems with their peers [2]. Faisal et al. studied problem-based learning at a medical school in Pakistan and found that students who learned through problem-based learning had improved content comprehension compared to lecture-based learning [18]. We believe that educators should consider incorporating problem or team-based learning activities into their online education.

Across the globe, undergraduate medical education has been dramatically impacted by the COVID-19 pandemic. In countries such as Pakistan, there are additional challenges faced by students and educators. This paper presents a focused discussion of the primary challenges affecting remote education, ranging from electricity to gaps in technology skill competencies. For each of these challenges, we presented specific solutions that institutions can consider. We also presented additional strategies for best practices in remote medical education. As the COVID-pandemic evolves, additional research is needed on the most effective educational strategies, especially for LMICs.

## Ethical approval

Not mandatory for this type of papers “Commentary”.

## Funding

None.

## Authors’ contributions

Z.Y, M.J.T, and H.S conceived the idea, H·S, M.J.T, Z.Y, A.N, and I·U retrieved the data, did write up of the manuscript, and finally, Z.Y, M.S.A, Z.D, and I·U reviewed and provided inputs. All authors approved the final version of the manuscript.

## Consent

N/A.

## Registration of research studies

1. Name of the registry: N/A
2. Unique Identifying number or registration ID: N/A
3. Hyperlink to your specific registration (must be publicly accessible and will be checked): N/A

## Guarantor

Muhammad Junaid Tahir: junaid262626@gmail.com, https://orcid.org/0000-0002-0335-6681.

## Declaration of competing interest

The authors have no conflict of interest.
